# Co-Variation between Seed Dormancy, Growth Rate and Flowering Time Changes with Latitude in *Arabidopsis thaliana*

**DOI:** 10.1371/journal.pone.0061075

**Published:** 2013-05-23

**Authors:** Marilyne Debieu, Chunlao Tang, Benjamin Stich, Tobias Sikosek, Sigi Effgen, Emily Josephs, Johanna Schmitt, Magnus Nordborg, Maarten Koornneef, Juliette de Meaux

**Affiliations:** 1 Laboratoire des Interactions Plantes-Microorganismes, CNRS UMR2594, Castanet-Tolosan, France; 2 Department of Plant Breeding and Genetics, Max-Planck Institute for Plant Breeding Research, Cologne, Germany; 3 Molecular and Computational Biology, University of Southern California, Los Angeles, California, United States of America; 4 Quantitative Crop Genetics, Max-Planck Institute for Plant Breeding Research, Cologne, Germany; 5 Institute for Evolution and Biodiversity, University of Münster, Münster, Germany; 6 Department of Ecology and Evolutionary Biology, Brown University, Providence, Rhode Island, United States of America; 7 Evolution and Ecology, University of California Davis, Davis, California, United States of America; 8 Population Genetics, Gregor Mendel Institute of Molecular Plant Biology, Vienna, Austria; 9 Laboratory of Genetics, Wageningen University, Wageningen, The Netherlands; The Australian National University, Australia

## Abstract

Life-history traits controlling the duration and timing of developmental phases in the life cycle jointly determine fitness. Therefore, life-history traits studied in isolation provide an incomplete view on the relevance of life-cycle variation for adaptation. In this study, we examine genetic variation in traits covering the major life history events of the annual species *Arabidopsis thaliana*: seed dormancy, vegetative growth rate and flowering time. In a sample of 112 genotypes collected throughout the European range of the species, both seed dormancy and flowering time follow a latitudinal gradient independent of the major population structure gradient. This finding confirms previous studies reporting the adaptive evolution of these two traits. Here, however, we further analyze patterns of co-variation among traits. We observe that co-variation between primary dormancy, vegetative growth rate and flowering time also follows a latitudinal cline. At higher latitudes, vegetative growth rate is positively correlated with primary dormancy and negatively with flowering time. In the South, this trend disappears. Patterns of trait co-variation change, presumably because major environmental gradients shift with latitude. This pattern appears unrelated to population structure, suggesting that changes in the coordinated evolution of major life history traits is adaptive. Our data suggest that *A. thaliana* provides a good model for the evolution of trade-offs and their genetic basis.

## Introduction

**Figure 1:**
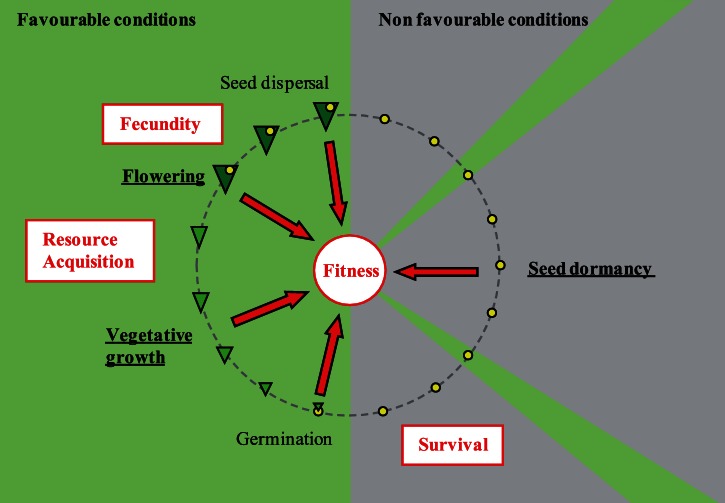
Major life-history traits and their effect on fitness in annual plant species. Green triangle: plant, yellow circle: seed, green sector: conditions favourable for growth, grey sector: adverse conditions. Red arrows show the participation of each trait to lifetime fitness, via their effect on survival, resource acquisition or fecundity.

The seasonal period favorable to growth and reproduction is geographically variable. In the North, the onset of spring is increasingly delayed, whereas in the South, increased risk of summer drought shortens the length of the growth period. The maintenance of populations across diverse climatic ranges might therefore require the coordinated evolution of major life history traits, such as germination, growth rate and flowering time, for a suitable synchronization of developmental phases with seasons. However, plant developmental traits are generally studied in isolation and few studies have investigated whether they evolve in concert [1]. Much can be learned from the study of genetic variation in the development of annual plants for two reasons. First, plants are directly exposed to adverse seasons and cannot chase favorable niches at any time of their development. Second, annual plant species complete their life-cycle in a single year and their fitness is determined by a single reproductive event. Therefore, selection on the synchronization of life-history decisions with the timing and length of the optimal season is expected to be particularly strong (Fig. 1).

The timing of germination imposes the conditions under which seedlings grow. It is therefore crucial to seedling survival and plant fitness [2], [3], [4], [5], [6], [7]. Germination is modulated by the environmental conditions after dispersal as well as by seed dormancy, a trait defined as the ability of a viable seed *not* to germinate in conditions favourable to germination [8], [9], [10], [11]. Dormancy thus contributes to delayed germination beyond adverse seasons, such as freezing temperatures in winter or prolonged periods of drought in summer. Two types of physiological dormancy can be distinguished: primary dormancy, which is established on the mother plant and secondary dormancy, which is established after dispersal, in response to environmental conditions that are not favourable for germination [12], [13], [14].

The timing of flowering affects the environmental conditions in which fertilization and seed maturation occur. Consequently, flowering time influences fecundity rate and plant fitness [15], [16], [17], [18], [19], [20]. It is controlled by various environmental signals experienced at both the seed and rosette stages [21], [22]. Finally, between germination and flowering, vegetative growth rate plays an important role as it determines the amount of resources that can be allocated to reproduction when flowering ends vegetative growth. An increased growth rate can potentially compensate for the limited size at maturity of early flowering genotypes but will decrease drought tolerance [23], [24], [25], [15].

Life-history traits jointly determine fitness and presumably evolve under trade-offs, but how trade-offs vary is poorly understood [26]. *Arabidopsis thaliana* offers an ideal context to study how adaptation influences life-history trait co-variation [27]. Since the last glaciation, this annual species has dramatically expanded its range and grows in climates with contrasting temperature and precipitation profiles [28]. Moreover, significant natural genetic variation of germination-related traits, vegetative growth-related traits and flowering time has been reported in this species [29], [30], [31], [32], [33], [34], [35], [36], [37]. Individually, these life history traits can influence fitness, although their respective impact on fitness can be variable [38], [6], [39], [40]. Germination, flowering time and vegetative growth rate follow environmental clines: germination appears to be increasingly delayed and flowering time accelerated as the adverse season imposes harsher selection on summer survival [41], [40], [42], [43], [36], [1], [44], [45]. However, co-variation with latitudinal or climatic gradients provides strong indication for adaptive evolution only when controlling for co-variation with population structure [46], [47], [40]. Indeed, the separation of populations during glaciations or after colonization of new geographical areas can also create latitudinal spatial structure in genetic variation, in the absence of adaptive evolution.

Recently, patterns of co-variation among various life-cycle traits were shown to follow a regional climatic cline in *Arabidopsis thaliana* populations of Northern Spain [48], [1]. To our knowledge, patterns of correlated evolution among the main life-cycle traits have not been studied at the continental scale, over the breadth of environments in which the species is found. The concerted evolution of life-cycle traits, however, is expected to depend on the nature and steepness of environmental gradients. Along a gradient in season length, like the one occurring in Scandinavia, natural selection for accelerated development might increase as the season shortens. In such context, populations selected for later flowering (presumably *via* increased vernalization requirement) could have been also selected for lower dormancy, in order to ensure immediate germination and allow rosettes to be large enough before winter outbreak. In this case, flowering time and dormancy are expected to be negatively correlated. By contrast, along an environmental gradient that increasingly favours multiple generations per year, early flowering will tend to be coupled with low dormancy to shorten the life-cycle, leading to a positive correlation between the two traits. Alternatively, in areas where the environment after seed dispersal is not permissive to germination, no selection pressure will operate on seed dormancy and flowering time can evolve independently. Therefore, various patterns of co-variation between dormancy and flowering time could be observed. The same may apply to patterns of co-variation between vegetative growth rate and germination or flowering time. Selection should act to increase growth rate where early flowering is advantageous. Growth rate, however, is also likely to decrease as the environment becomes drier because active growth requires intensive gas exchanges and thus enhances water loss [27]. In conclusion, as the environmental changes, evolutionary trade-offs between life-cycle traits may shift, or even disappear. This has been documented with both simulation and experimental data in insects [49], [50]. But, surprisingly, the idea that trait co-variation may change across environments has attracted little attention in studies of plant life-history traits.

In this study, life-history traits covering the whole life cycle, i.e. primary seed dormancy, secondary seed dormancy, vegetative growth rate and flowering time, were characterized in greenhouse conditions for 112 *A. thaliana* genotypes sampled across the breadth of the European latitudinal range of the species. The genetic basis of each of these traits was reported previously in a Genome-Wide Association (GWA) mapping study [51]. Here, we bring further evidence for their adaptive relevance by showing that species-wide genetic variation in flowering time and primary dormancy follows a latitudinal gradient, independent of the population structure gradient. Importantly, we show that traits do co-vary, but their pattern of co-variation is complex and changes with latitude. We interpret this finding as a result from latitudinal shifts in the nature of environmental gradients, which modulate the relative strength of selection pressure on the components of the life-cycle. We conclude that trait co-variation in *Arabidopsis thaliana* likely depends on the geographical scale considered and on the nature of the environmental gradient associated.

## Materials and Methods

### Plant material and environmental gradient

**Figure 2:**
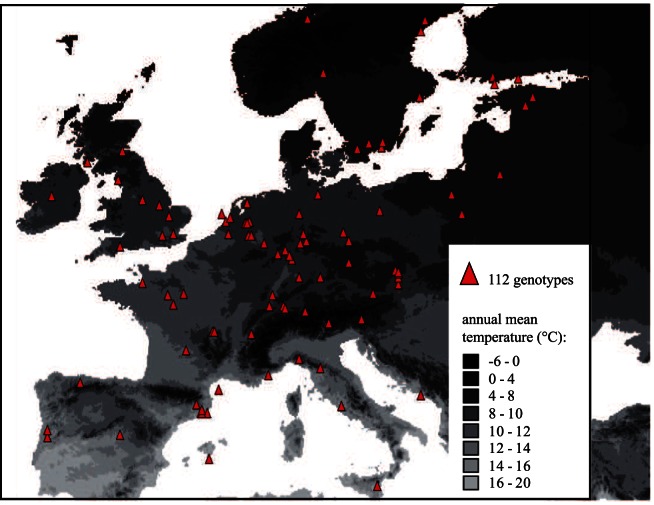
Location of origin the 112 European genotypes used in this study.

A total of 112 European *A. thaliana* genotypes obtained from the ABRC and NASC stock centers were used for the study (Table S1, Fig. 2). The location of origin of these genotypes ranges from 37.5° to 63.3° (decimal degree) in latitude and from −8.3° to 26.0° (decimal degree) in longitude (Table S1, Fig. 2). We focused on European accessions because, at given latitude, the climate experienced by non-European genotypes generally differs from the one experienced by European. Genotypes originating from the most Northern part of Scandinavia are believed to have a markedly different population history and were not included in the sample [52]. Each accession was grown in four replicates and growth rate, flowering time and seed dormancy were assessed as described below.

### Measurement of vegetative plant growth rate

After stratification at 4°C for two days (water imbibed seeds on filter paper in Petri dishes), seeds were planted in potting soil, grown for one week in the greenhouse under long day (with supplementary light maintaining long day conditions), vernalized for 4 weeks (4°C, 16 h light, 50% relative humidity) and subsequently placed back and randomized into the greenhouse until silique maturation. To estimate vegetative growth rate, we used a non-destructive method based on digital imaging described earlier in [53], [54]. Plants were photographed from above with a charge coupled device (CCD) camera (Sony DSC-F828) on the last day of the vernalization treatment, as well as after one week of growth in the greenhouse after vernalization (at that time all genotypes were still in vegetative growth phase). Leaf area at these time points was calculated using Image Pro Analyzer 6.0 (MediaCybernetics) in cm^2^ and vegetative growth rate was estimated as the increment of cm^2^ leaf area per day between the last day of cold treatment and the 7^th^ day of growth in greenhouse. Estimates of growth rate in the greenhouse were correlated to estimates of growth rate during vernalization, *i.e.* on smaller rosette growing at 4°C (*R^2^* = 0.77, p<0.001, not shown).

### Measurement of flowering time

Flowering time was scored as number of days from sowing the seeds in soil to the day that the petals of first flower were visible. The total number of leaves at bolting was also measured and correlated strongly with flowering time (not shown). At the end of this experiment, matured siliques of approximately equal age were collected in bags for each individual plant for seed dormancy measurements. After harvest, seeds were stored in laboratory conditions in paper bags.

### Measurements of germination-related traits

All measurements of primary and secondary dormancy, for each genotype, were conducted with four independent seed batches (each batch contained seeds from one plant) collected from the experiment described above. Primary dormancy was measured as the progressive increase of germination rate measured after 7, 28, 56, 91, 133, 182 and 209–269 days of dry storage in laboratory conditions. This procedure is defined as after-ripening and is described in [55]. The germination percentage at each time point was determined by counting how many of approximately 70 seeds had germinated after one week of imbibition in growth chamber (25°C 12 h day/ 20°C 12 h night). After approximately 9 months, the experiment was stopped and the viability of remaining non-germinating seeds was confirmed by provoking germination with a mixture of 100-*μ*M gibberellin GA_4/7_ (ICI Ltd, Bracknel, UK) and 38-*μ*M fluridone (Dow Chemical Co., Hitchen, UK). Both fluridone and GA_4/7_ were initially dissolved in ethanol and then diluted. The final concentration of ethanol was less than 0.03% as described in [14].

Dormancy was quantified as the Duration of Seed Dry Storage required for reaching 50% of seed germination (DSDS_50_) as defined in [55]. Dormancy release followed different dynamics over time that could not be described by a single model. DSDS_50_ was therefore simply estimated in number of days, by extrapolating from a straight line between the two time points at which less and more than 50% seeds germinated.

Primary dormancy is an environment-dependent trait. To validate the genetic robustness of our phenotypic data, we thus performed three independent experiments. The first experiment was conducted with 3 replicates of the same set of genotypes, grown in a different greenhouse and tested for dormancy release in the way described above. A second experiment was conducted on a subset of 52 genotypes grown in environmentally controlled growth chambers MTPS72 from Conviron, Canada (75% relative humidity, 18°C, 8 hrs of light/16 hrs dark). Moreover for a subset of 22 genotypes, germination rates of seeds harvested from a common garden experiment in the field (in Valencia, Spain) were also scored after 2 months after-ripening. Since this measurement was done at only one time point, DSDS50 could not be calculated. A lower germination rate is expected for genotypes showing stronger dormancy. In this last experiment, since seeds were collected from field grown plants, variation in maternal environments during seed maturation was not controlled for. No specific permissions were required for the described field study, which did not involve endangered or protected species. The location of the field study is not private or protected in any way. In total, the characterization of seed dormancy variation required scoring the percentage of germination in more than 10 000 Petri dishes.

To measure secondary dormancy, only genotypes (79) that displayed more than 85% of germination at the end of primary dormancy measurement (fully after-ripened seeds) were used. This allowed not confounding residual primary dormancy from cold-induced secondary dormancy. Secondary dormancy was estimated as the reduction in germination rate of fully after-ripened seeds after a 6-week long exposure to 4°C in darkness. For this, seeds were imbibed in laboratory conditions (21°C, light) with 500 µl of sterile water, on sterile filter paper, in sterile petri dish and in flow hood. The experiment was conducted in sterile conditions to minimize fungal contaminations, which can complicate germinant counting, although seeds themselves were not sterilized. Petri dishes were closed with parafilm, wrapped in aluminum foil to ensure total darkness and placed in a chamber at 4°C for six weeks. Subsequently, cold-treated seeds were placed for germination in the 25°C 12 h day/20°C 12 h night chamber for one week, thereafter germination was scored as described in [14]. Secondary dormancy was given by the absolute value of the slope (percent of germinants per day) between the germination percentages of the fully after-ripened seeds and after six weeks of cold treatment; the higher the absolute value of the slope, the higher the proportion of seeds entering secondary dormancy. Viability of non-germinated seeds after cold treatment was confirmed as described in above for primary dormancy.

### Population genetic structure gradient

To disentangle the putative effect of demography and natural selection on trait variation, we investigated whether a gradient of population structure is detectable in our sample of genotypes. The plants were genotyped for a set of 149 single nucleotide polymorphism (SNP) markers described in [56] by Sequenom, inc. (San Diego, CA). Out of the 149 SNP markers, 139 were polymorphic in the whole sample and showed a proportion of missing data of 0.04. Based on these 139 SNPs, we used the Software structure2.2 [57] to assess the existence of population structure in our sample. We used a haploid setting and the “linkage model” with “correlated allele frequencies”. The algorithm was run with a burn-in length of 200,000 MCMC iterations and then 100,000 iterations for estimating the parameters. This was repeated five times for each *K* (ranging from 1 to 20). The number of clusters (K) in our sample was detected as described in [58] via calculation of L(K) and ΔK. (Fig. S1).

### Statistical analyses

#### Variation and broad-sense heritability of life history traits

To determine whether genetic variation of each life history trait is significant within the whole sample of 112 genotypes with 4 replicates for each, an analysis of variance (ANOVA) with genotypes as random factor was performed using SYSTAT 11. In addition, for each trait, the broad-sense heritability (H^2^  =  genotype variance component/(genotype variance component + error variance component)) was calculated. Finally, to analyze genetic variation of life history traits, the adjusted entry means for all genotypes and for all traits were calculated using ASReml (http://cran.r-project.org).

#### Multivariate and uni-variate analyses of life cycle trait variation

To examine how life-history traits vary along both gradients of latitude and population structure, we conducted a multivariate analysis with the help of the R function adonis (Vegan Package, http://cran.r-project.org/). This approach was possible because only two populationstructure clusters were detected, so that population structure could be described by only one parameter (see below). The adonis function is essentially identical to a MANOVA analysis, but since it establishes p-values by permutation, it can better account for possible skews in the distribution of the variables. The model included the matrix of life-history traits as dependent variables with latitude, population structure (output by STRUCTURE with K = 2, see above), and the interaction between the two gradients as independent co-variates. The matrix of life history traits included primary dormancy, vegetative growth rate and flowering time. Secondary dormancy entailed a larger number of missing data. The multivariate analysis was therefore performed with and without including secondary seed dormancy. A classical MANOVA, which assumes a normal distribution of the traits, yielded the same results (not shown). Uni-variate analyses were performed using the *lmp* function (lmPerm Package, http://cran.r-project.org/) with vs. without latitude as independent co-variate. To establish the significance of one co-variate (e.g. latitude), we used an F-test comparing a full model (including both covariates and their interaction) to a model with only the other co-variate (e.g. population structure). The difference of the multiple R-squared values between the two models was used to estimate the percentage of variance of the dependent variables explained by latitude. The interaction between the two co-variates was not significant for any of the four traits.

#### Correlations between life history traits

The covariation of life-cycle traits was quantified with Pearson genetic correlation coefficients. Pearson correlation coefficients were calculated for each pair of traits for the whole sample of 112 genotypes. Nevertheless, over the whole sample, some correlations might be masked and appear only among genotypes distributed in a small latitudinal window. Indeed, the nature of environmental gradients can change with latitude. To assess how latitude modifies the co-variation between pairs of traits, we pursued two approaches. First, we tested for an effect of pairwise trait interaction along the latitudinal gradient, with latitude as dependent variable, and two life-cycle traits as independent main effects and interaction covariates. Here again, to account for possible skews in the distribution of the variables, we used the R function lmp, which establishes p-values by permutation. Second, correlation coefficients were calculated from subsamples of genotypes along a sliding latitudinal window including 45 genotypes. To test whether correlation coefficients within window co-varied significantly with latitude, we generated 1000 datasets with permuted latitude and computed R^2^ between latitude and trait correlation for each permutation. Significance was quantified by calculating the proportion of permuted datasets showing greater R^2^ values. The analysis was also performed for sliding windows of 35 or 25 individuals and showed the same trend, although p values weakened as the number of individuals in the window decreased (not shown).

## Results

### Natural genetic variation in life history traits

**Figure 3:**
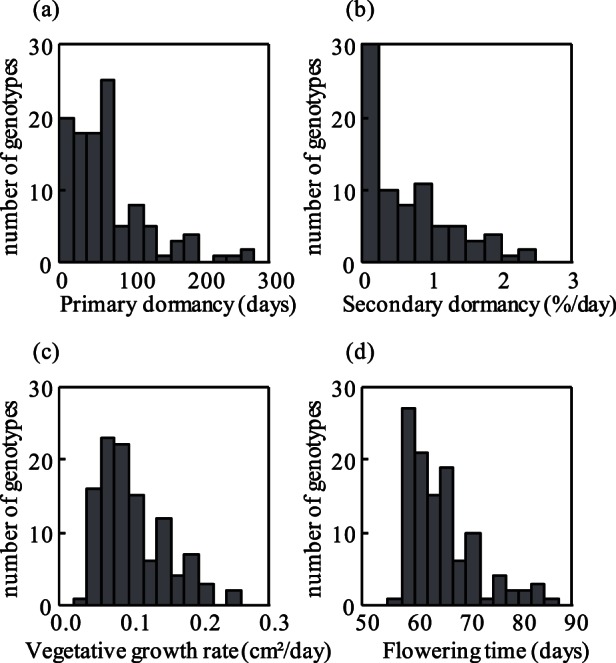
Histograms showing natural genetic variation of life history traits using adjusted means. (a) Primary dormancy (days): measured by number of days required to reach 50% of germination (DSDS50). (b) Secondary dormancy (% of germination decrease per day): measured by the reduction in germination rate of fully after-ripened seed after a 6-week long exposure to 4°C in darkness. (c) Vegetative growth rate (cm^2^/day): measured by increase of leaf area during one week in the greenhouse. (d) Flowering time (days): number of days until opening of the first flower.

Among a sample of 112 European *A. thaliana* genotypes, total phenotypic variance (primary dormancy, secondary dormancy, vegetative growth rate and flowering time after vernalization) was significantly explained by the genotype (Table 1). Estimates of broad-sense heritability were all of 0.84 or greater and adjusted means were calculated for each genotype (Fig. 3, Table 1).

**Table 1:** ANOVA table reporting significant effect of the genotype on phenotypic variance for each life-history traits and associating broad-sense heritability *H^2^*.

	Genotype			Error		
Trait	df	Mean squares	*F*	df	Mean squares	*H^2^*
Primary dormancy	110	13272.6	11.12***	322	1193.7	0.92
Secondary dormancy	78	1.52	8.55***	225	0.18	0.89
Vegetative growth rate	110	0.0089	5.43***	308	0.0016	0.84
Flowering time	111	171.99	38.30***	328	4.49	0.97

df: degree of freedom. ***p<0.001.

The release of primary dormancy (DSDS50) was generally monotonous but occurred at variable speed (Fig. 3a). DSDS50 ranged from 3.5 to 264 days, with a median value of 60 days (Fig. 3a). This experiment was replicated independently with another batch of seeds harvested from greenhouse grown plants, where DSDS50 ranged from 3.5 to 79 days, with a median value of 15 days. Although measurements of dormancy were generally lower and the overall variance was more restricted in this independent experiment, the data from both experiments were correlated (r = 0.51, *P*<0.001). We focused on the first dataset because it allowed the best differention among weakly dormant genotypes. Correlation coefficients of 0.5 across experiments seem, however, to be quite typical for seed dormancy, presumably because it is a highly plastic trait strongly affected by the maternal environment [59]. In a subset of 52 genotypes, seed dormancy was measured independently using seeds grown in a growth chamber experiment under uniform light and temperature conditions. This yielded similarly correlated results (r = 0.49, *P*<0.001). Eventually, for another subset of 22 genotypes, germination rates of seeds after ripened for approximately 2 months after harvest in the field (in Valencia, Spain) were scored. As expected, the genotypes that were most dormant in the greenhouse experiments (high DSDS_50_), showed the lowest germination percentage when matured in the field (r = −0.5, *P* = 0.02).

We also found high heritability and significant genetic variation in secondary dormancy (*H^2^* = 0.89, *F_78,225_*  = 8.55, *P*<0.001, Table 1 and Fig. 3b). Secondary dormancy ranged from 0 (no secondary dormancy) to 2.28 (percentage germination decrease/day) with a median value of 0.4, which corresponds to a 16% decrease in germination percentage. After this prolonged exposure to cold, some genotypes, such as Baa-1, Sav-0 and Est-1, showed a particularly strong response, with a germination percentage decreased by more than 90%.

The total phenotypic variance of vegetative growth rate was also significantly explained by genotypes (*F_110,308_*  = 5.43, *P*<0.001). Heritability of vegetative growth rate was high: 0.84 (Table 1 and Fig. 3c). The median for this trait was 0.095 (cm^2^/day). The rosette leaf area of the fastest growing genotypes, Be-0 or Bay-0, grew ten times faster than the slowest growing genotype HR-5.

Finally, significant genetic variation was also detected for flowering time (*F_111,328_*  = 38.3, *P*<0.001). Among the traits described in this study, flowering timing showed the highest heritability: 0.97 (Table 1 and Fig. 3d). As the vernalization treatment accelerated flowering, the distribution of genetic variation of flowering time was skewed towards early flowering with a median of 63 days.

### Population structure in Europe follows an East-West gradient

The analysis of SNP variation across the genome revealed that two major genetic groups (K = 2) divide the sample (Fig. S1). The correlation between population structure (probability to belong to one of the two genetic clusters) and latitude was not significant (r = 0.15, p = 0.10). By contrast, the correlation with longitude was highly positive and significant (r = 0.71, p<0.001) indicating that, in the sample studied here, population structure follows a longitudinal gradient, independent of the latitudinal gradient.

### Latitude gradient influences multivariate life-cycle variation

**Figure 4:**
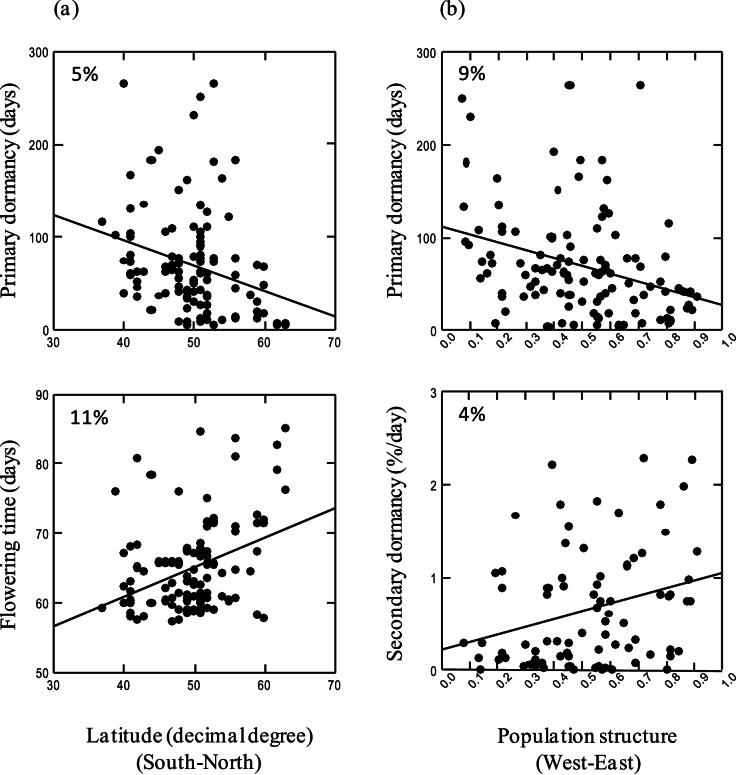
Life history traits as a function of the gradients in latitude (expressed in °North) and/or population structure (measured as the relative contribution to the first population structure group). Only significant effects reported in Table 2 are shown with the percentage of variance explained associated. (a) Latitude has a significant effect on primary dormancy and flowering time, p<0.05 and p<0.001, respectively. (b) Population structure estimated by the probability to belong to one of the two clusters, has a significant effect on primary and secondary dormancy, p<0.01 and p<0.1, respectively.

We performed both multivariate and univariate analyses to assess the relationship between genetic variation of life history traits and the two gradients: latitude and population structure. The multivariate analysis revealed a marked effect of latitude on life-history trait variation, and a milder effect of the population structure gradient (Table 2, R^2^ = 0.11 and 0.09, respectively, both *p*<0.001). Results were similar when secondary dormancy was included (Table 2). Both primary dormancy and flowering time varied strongly with latitude (Table 2, both p<0.0001). Overall, plants tended to flower later in the North and earlier in the South (Fig. 4). The level of primary dormancy tended to be lower in the North and higher in the South (Fig. 4). Clinal variation in dormancy was also recently reported in *Beta vulgaris ssp. maritima* [60]. Nevertheless primary dormancy contrary to flowering time was significantly dependent on the gradient of population structure (Table 2). By contrast, secondary dormancy was only weakly influenced by the population structure gradient (p<0.1, Table 2). Notably, primary and secondary dormancy tended to follow the population structure gradient in opposite direction (Fig. 4). No effect of latitude or population structure was detected for growth rate (Table 2). In conclusion, since population structure was included in the model and is unrelated to latitude, our results suggest the existence of an adaptive cline for two traits: primary dormancy and flowering time (Fig. 4).

**Table 2:** Multi- and uni-variate analyses of life-history variation, p-values associated with the effects of latitude and population structure.

		Latitude		Population structure	
Trait	df	*F*	% Explained variance	*F*	% Explained variance
**Multivariate analysis**					
(excluding secondary dormancy)	1,109	16.06***	11%	12.51**	9%
(including secondary dormancy)	1,75	22.01***	20%	7.79**	7%
**Univariate analysis**					
Primary dormancy	2,107	4.56*	5%	6.99**	9%
Secondary dormancy	2,75	0.32	n.s.	2.66•	4%
Vegetative growth rate	2,107	1.29	n.s.	2.16	n.s.
Flowering time	2,108	7.95***	11%	0.16	n.s.

Interaction between the two gradients was not significant (not shown). Because of missing values in secondary dormancy, the multivariate analysis was conducted with and without secondary dormancy. P-values were established with F test based on permutations (see methods). n.s.: not significant, • p<0.1, * p<0.05, ** p<0.01, ***p<0.001. The percentage of variance explained by latitude or population structure gradients is given. df: degrees of freedom for numerator and denominator, respectively.

### Correlations between life history traits within the sample of genotypes

**Figure 5:**
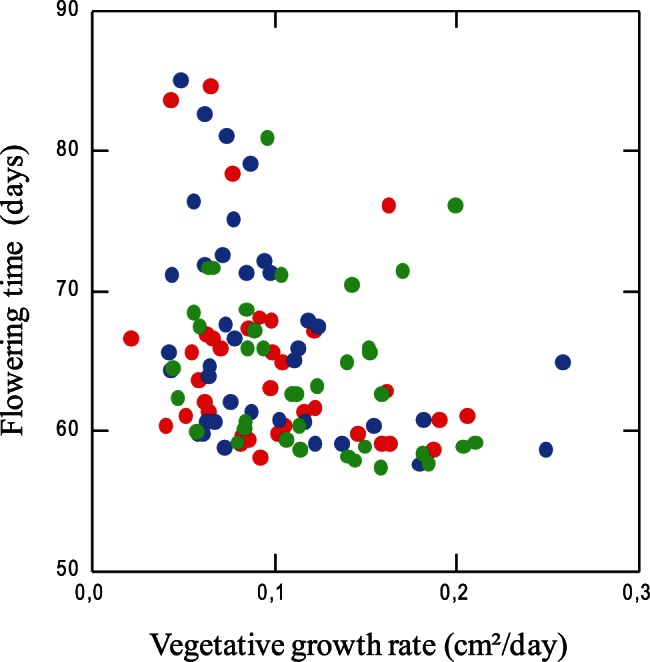
Flowering time as a function of vegetative growth rate. The 112 European genotypes were divided in three categories: low (blue dots), medium (green dots) and high primary seed dormancy level (red dots).

If the components of the plant life-cycle evolve in concert via trade-offs or correlated evolution, genetic correlations among traits should arise. Overall, the genotypes, which flowered late, tended to grow more slowly and express weaker primary seed dormancy whereas earlier flowering genotypes tended to grow faster and express a higher dormancy level (Fig. 5). We calculated Pearson correlation coefficients simultaneously for all pair of traits and included Bonferroni correction to correct for multiple testing (Table 3). Within our European sample, analyses of co-variation between traits revealed that only vegetative growth rate and flowering time co-varied significantly (r = −0.32; p = 0.002). We observed that, two genotypes (Mr-0, from Italy and Omo2-1, from Sweden) expressed an outlying trait combination with strong primary dormancy and late flowering (Table 3 and Fig. 5). Finally, there was no significant correlation between primary and secondary seed dormancy (r = −0.06; p = 0.56). This pattern of trait co-variation was not dependent on the presence of a functional allele at the *FRIGIDA* locus (not shown; [61]). However, our sample is too small to address this question properly.

**Table 3:** Pearson correlation coefficients between life history traits (112 genotypes).

	Primary dormancy	Vegetative growth rate	Flowering time
Primary dormancy		1.00	0.38
Vegetative growth rate	0.077		0.002
Flowering time	−0.15	−0.32	

Coefficient of correlation (*r*) and the Bonferroni corrected p-values are shown below and above the diagonal, respectively. Secondary dormancy is not correlated with other traits (not shown).

### Changing correlation between life-history traits along the latitudinal gradient

We hypothesized that changes in natural selection pressure along the latitudinal gradient may influence the extent to which life-history traits evolve in concert. Indeed, in a geographically heterogeneous environment, correlated selection on multiple traits could vary, leading to changing patterns of trait correlations. Therefore trait co-variation should be analyzed in function of an environmental gradient. This may reveal patterns of trait variation that were masked in the analyses reported above. We first tested the effect of interaction between trait pairs on latitude using a linear model. Latitude had a significant effect on each of the three pair-wise interactions between primary dormancy, flowering time and growth rate, whereas interactions between secondary dormancy and all other traits were unaffected by latitude (Table 4). Note that since the latitudinal and population structure gradients were not correlated, this effect remained significant even if population structure was introduced in the model (not shown).

**Table 4:** Pairs of trait with a latitudinal gradient of co-variation.

	Primary dormancy	Secondary dormancy	Vegetative growth rate	Flowering time
Primary dormancy		-	3%	7%
Secondary dormancy	0.267		-	-
Vegetative growth rate	0.032	0.111		6%
Flowering time	<0.0001	0.441	0.005	

Significance was tested with a linear regression model, with latitude as a dependent variable (see methods). p-values associated to the interaction between the traits and the percentage of the latitudinal variation explained by the interaction are shown below and above the diagonal, respectively.

**Figure 6:**
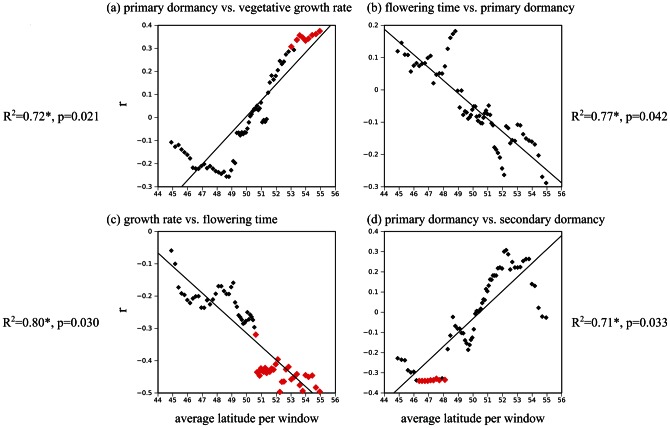
Pairwise trait correlation between primary dormancy, flowering time or growth rate change significantly along the latitudinal gradient. The correlation coefficient *r* (−1<*r*<1), on the y-axis, was calculated along a sliding latitudinal window with a fixed number of 45 genotypes. The average latitude of each 45-genotype window is given on the x-axis. Red points show local r values with associated *p*<0.05. The change in pairwise trait correlation *r* with latitude was quantified by the regression coefficient R^2^, and its associated *p-value* (see methods). With the exception of (d), results hold when sliding windows encompass 25 or 30 genotypes.

The approach above, however, was unorthodox, because to differentiate the effect of individual traits from their co-variation, latitude had to be considered as dependent variable in the linear model (see methods). We therefore took a second approach to illustrate the effect of latitude on trait co-variation: coefficients of correlation between pairs of phenotypes were calculated across sliding latitudinal windows encompassing a fixed number of 45 genotypes (Fig. 6). This representation allows highlighting windows with a significant correlation. Thousand datasets with permuted latitudes of origin provided a non-parametric way to determine the significance of the observed pattern. The correlation between average growth rate and primary dormancy increased significantly with latitude (R^2^ = 0.72, p = 0.021) and reached significance at high latitudes only (Fig. 6-a). The two traits tended to be negatively correlated at Southern latitudes whereas in the North, individuals with greater dormancy showed higher growth rate and individuals with lower dormancy showed lower growth rate (Fig. 6-a). The correlation between flowering time and primary dormancy, instead, showed a significant tendency to decrease with latitude (R^2^ = 0.77, *p* = 0.042, Fig. 6-b), although the local correlation was never sufficiently strong to be significantly different from zero (Fig. 6-b). In the South, individuals with weaker dormancy tended to flower earlier (rapid cycling) whereas in the North, individuals with weaker dormancy tended to flower later. The correlation between growth rate and flowering time also decreased with latitude, being significantly negative at the highest latitudes (R^2^ = 0.80, *p* = 0.03, Fig. 6-c). Surprisingly, the correlation between primary dormancy and secondary dormancy increased significantly with latitude (R^2^ = 0.71, p = 0.033, Fig. 6-d), although no effect was detected with the linear model (Table 4). The correlation was significantly negative among genotypes of Southern origin (Fig. 6-d). The discrepancy between the results of the linear model and the sliding window for this pair of traits may be caused by a larger amount of missing data for secondary dormancy. Indeed, strongly dormant genotypes with residual dormancy at the end of the primary dormancy analysis could not be assessed for secondary dormancy (see Methods). Such genotypes are over-represented in the South and under-represented in the North. In fact, this was the only pattern of co-variation that was lost when the size of the latitudinal window was decreased (not shown). Nevertheless, when the whole sample was partitioned in accessions originating from latitudes lower and greater than 51, the median latitude for genotypes with measurable secondary dormancy, correlation between primary and secondary dormancy was significant only for the southern group of accessions (r = −0.34, p = 0.023, vs, r = 0.18, p = 0.29), confirming the sliding-window analysis.

## Discussion

Life-history traits controlling the duration and timing of each life-cycle phase tend to co-vary due to both natural selection and genetic or physiological constraints [15], [62], [6], [39], [63], [26], [48], [1], [22]. Therefore, life-history traits studied in isolation provide an incomplete view on the relevance of life-cycle variation for adaptation. In this study, we examine genetic variation in primary dormancy, secondary dormancy, vegetative growth rate and flowering time of European *Arabidopsis thaliana* genotypes collected at diverse latitudes.

Natural environments are obviously more complex and often harsher than in the greenhouse. The genotypes in this study may express life-cycle traits differently in their environment of origin. Studies in greenhouses of a large sample of genotypes face this inevitable caveat. Nevertheless, significant genetic variation and high heritability was found in this study for all traits. We show in the following that although the genetic variation described here cannot be used to predict life-cycle in the field, it is suitable for the analysis of patterns of genetic co-variation and allows an ecological interpretation.

### Phenotypic variation in the lab *vs*. field

Although realized germination in native *A. thaliana* populations is not known, our measure of seed dormancy may help understand germination as it is expressed in the field. Indeed, primary seed dormancy measured in comparable conditions was reported to be under the control of the same QTLs as germination in the field [64]. In this study, levels of primary dormancy were also correlated with germination of seeds matured in the field (in Valencia, Spain). In addition, secondary dormancy triggered by cold exposure is controlled by several QTLs and is known to occur in the field [65], [66], [14], [67]. To the best of our knowledge, this study is the first to report on natural genetic variation for secondary seed dormancy in response to a prolonged exposure to cold. We have identified a number of genotypes that can trigger strong secondary dormancy. It will be interesting to test in future studies whether these genotypes would also trigger strong dormancy in the field.

Natural genetic variation of a large number of traits related to vegetative development was reported in *A. thaliana* both in the greenhouse and in the field [31], [32], [40], [68], [48]. In this study, we also confirm the presence of extensive genetic variation controlling the rate of rosette growth in *A. thaliana* plants subjected to conditions favorable to growth. Although here again, growth may occur at a different rate in the field, the genetic differences observed here are likely to also manifest at other temperatures because our rates of rosette growth were correlated to rates of rosette growth in the vernalization growth room (not shown).

Flowering time being under the control of multiple pathways, it is also difficult to predict flowering time in the field [37]. Genetic variation of flowering time has been extensively examined in greenhouse conditions [30], [69] and in the field [37], [70]. Yet, the genes controlling variation of flowering time differ largely between environments [70], [71]. In our experiment, flowering time was measured after a brief exposure to cold. As it did not equally satisfy the vernalization requirement of the genotypes, much of the genetic variation was still detectable [35]. The observed variation does not allow distinguishing between the ability to reach maturity fast and the magnitude of the plastic response to cold exposure. Nonetheless, we show below that the flowering time, as well as dormancy and growth rate variation reported in this study show signs of adaptive evolution.

### Clinal variation in dormancy and flowering time

Covariation between phenotypic traits and latitude can suggest adaptation when co-variation with population structure is being accounted for [46], [47], [40], [72]. We excluded non-European genotypes as well as European accessions from the extreme north of Scandinavia that were reported to have a very distinct demographic history [52]. In our sample of 112 European accessions, population structure was therefore reduced and displayed only an East-West gradient that was also observed in several previous studies [73], [74], [75]. This genetic differentiation between Eastern and Western Europe is thought to be the result of post-glacial recolonization from different refugia notably central Asia and the Iberian Peninsula [74], [73].

Confirming previous reports, we find that primary dormancy and flowering time follow a latitudinal cline [40], [48], [41], [60]. In the Iberic Peninsula, more than 25% of flowering time variation was explained by the altitudinal cline [44]. By comparison, the latitudinal clines detected here are modest, explaining not more than 11% of the phenotypic variance. The possibility that they reflect an undetected gradient of population structure cannot be fully excluded. Nevertheless, evidence for local adaptation across the latitudinal range is manifold [76], [77], [78]. The latitudinal gradient can thus be considered as a proxy for the environmental variation to which the genotypes included in this study have adapted. Indeed, in Europe, latitude correlates with day length, mean precipitation and mean temperatures.

Attempts to relate the clines observed here to specific environmental parameters, however, were not fruitful: we could not identify a climatic parameter explaining the data better than latitude itself (not shown). In fact, monthly or yearly average climatic parameters are not equally relevant to plant development in different locations. For example, mean temperature in February might be ecologically relevant for *A. thaliana* genotypes found in Spain but not in Northern Scandinavia where plant development still pauses. Identifying key ecological parameters important for local plant development requires that climatic information be properly calibrated on the progression of the growth season of the plant and not only on the calendar [79]. In Northern Spain, an altitudinal cline was observed for flowering time and germination traits, which was explained by a complex gradient in temperature and precipitation [48], [1]. Altitude indeed seems to cause a strong adaptive cline in flowering traits across the whole region [44]. In our sample, however, altitudinal data has not been reliably recorded for all genotypes, and the existence of an altitudinal cline could not be correctly assessed.

### Changes in co-variation between traits may result from latitudinal shifts in the nature of environmental gradients

The main novelty of this study, however, is not the report of clinal variation for individual traits but for trait covariance. We find that the interaction between traits can explain up to 6% of the latitudinal gradient (Table 4), a proportion comparable to that explained by the gradient of seed dormancy alone (Table 2). This analysis, in fact, shows that a trait like plant growth rate varies with latitude but only *via* its co-variation with other life-history traits. Analyses of co-variation using smaller samples along latitude (Fig. 6) show that significant correlations between traits can be masked if the full sample is the only one considered (Table 3).

For example, at first sight, we observed no significant co-variation between vegetative growth rate and latitude (Table 2). However, our analysis reveals that growth rate varies with latitude *via* its co-variation with primary dormancy and flowering time (Table 4, Fig. 6a–c). Correlations with growth rate can be locally significant or even shift in sign across the whole range (Table 4 and Fig. 6). To the best of our knowledge, such changing patterns of co-variation have not been reported in annual plants. This possibly results from the analysis of trait co-variation over a breadth of environments represented by the latitudinal gradient.

In the North, growth rate shows locally significant negative co-variation with flowering time and positive co-variation with dormancy (Fig. 6a–c). Genotypes that are the most dormant tend to grow faster and flower earlier. This combination of traits is in agreement with the idea that shorter season length (i.e. late spring onset, fast reduction of day length in the fall, and increased vernalization requirement) imposes limitations on the time that can be spent at the seed stage. The negative correlation between flowering time and growth rate further suggests that growth rate may be selected to alleviate the trade-off between flowering time and plant size at maturity [15]. In the Southern areas, however, increased growth rate does not associate with either increased earliness, or increased dormancy (Fig. 6a, c). This may suggest that it is not flowering time *per se*, but the vernalization requirement that co-varies with growth rate and dormancy in the North. Indeed, the brief exposure to cold may have reduced the magnitude of flowering time differences in the South only, where vernalization requirements are weaker [35]. Alternatively, it would be tempting to speculate that this pattern results from stronger negative consequences for drought resistance entailed by faster growth in the South [53], [24].

As for vegetative growth rate, secondary dormancy, taken in isolation, did not show significant co-variation with latitude (Table 2). Intriguingly, the correlation between primary and secondary seed dormancy was locally significant at lower latitude (Fig. 6d), although changes in trait correlation was not significantly associated with latitude (Table 4). Genotypes showing stronger primary dormancy tended to have weaker secondary dormancy in response to low temperature. In Spanish populations, primary dormancy and secondary dormancy were shown to have a complementary action on the response to spring vs. fall germination cues: genotypes tended to express a similar germination behavior via either primary or secondary dormancy [1]. Although in the latter study, secondary dormancy was induced not by low but by high temperature, our analysis suggests that the coordinated action of primary dormancy and secondary dormancy on germination preferences may be regional. This result nonetheless should be taken with caution because secondary dormancy could not be measured for the most dormant genotypes, which may have biased the pattern of trait correlation.

## Conclusion

Our study shows that trait co-variation in *A. thaliana* depends on the geographical region considered and presumably on the nature of the environmental gradient it covers. While there is growing evidence that life-cycle traits co-vary [48], [1], this is, to the best of our knowledge, the first time that changing patterns of co-variation are reported in annual plants. The pattern observed is independent of the gradient in population structure, suggesting that shifts in trait correlation have an adaptive relevance. In the field, the timing of flowering was shown to depend on the timing of germination [37]. Our study now confirms that understanding genetic variation in individual life-history traits requires an understanding of genetic variation controlling the other components of the life cycle. Trade-offs play an important role in constraining the evolution of life-history traits, but little is known about their evolution [26]. This study now suggests that detecting trade-offs will depend on the geographical scale considered and on the nature of the environmental gradient associated. Genetic associations between single life-history traits and the alleles segregating at major QTLs change across the species range [35], [44], [41]. In the future, identifying the genetic basis controlling changes in trait correlations [80] promises to help understand and, may be, even predict the evolution of life history strategies.

## Supporting Information

Figure S1 Population structure and detection of the true number of clusters (K) via graphical method.
(PDF)

Table S1 Stock number, name, country, longitude, latitude, phenotypes and population structure of the 112 European genotypes used in this study.
(PDF)
