# Supplementary figures and images for: Co-Variation between Seed Dormancy, Growth Rate and Flowering Time Changes with Latitude in Arabidopsis thaliana
Source: PLoS One. 2013 May 23;8(5):e61075. doi: 10.1371/journal.pone.0061075 (PMC3662791; doi:10.1371/journal.pone.0061075)

Fig. S1 Population structure and detection of the true number of clusters (K) via graphical method

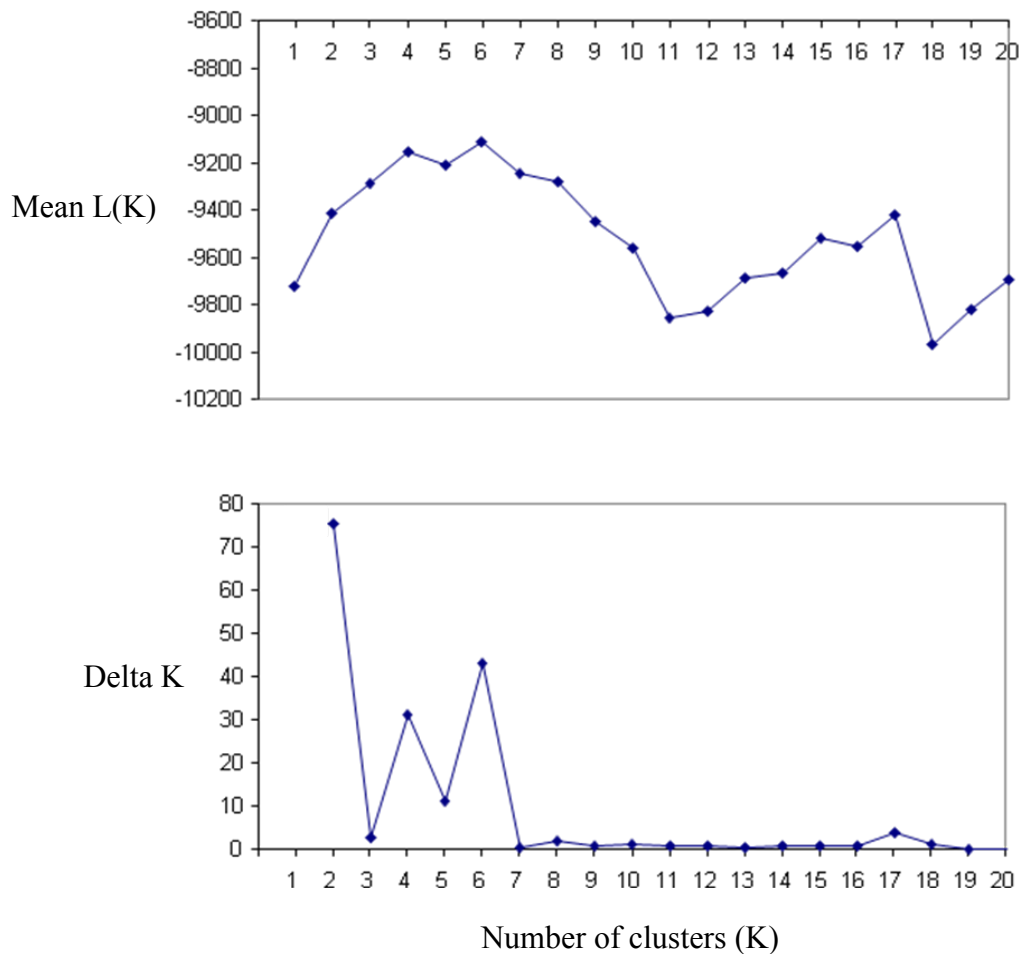

Supplement: Figure S1 — Population structure and detection of the true number of clusters (K) via graphical method. (PDF) [file pone.0061075.s001.pdf]
