# Supplementary material for: Co-Variation between Seed Dormancy, Growth Rate and Flowering Time Changes with Latitude in Arabidopsis thaliana
Source: PLoS One. 2013 May 23;8(5):e61075. doi: 10.1371/journal.pone.0061075 (PMC3662791; doi:10.1371/journal.pone.0061075)
Supplement: Table S1 — Stock number, name, country, longitude, latitude, phenotypes and population structure of the 112 European genotypes used in this study. (PDF) [file pone.0061075.s002.pdf]

| Stock Number (CS) | Name | Country | Longitude | Latitude | Primary dormancy-rep1 | Primary dormancy-rep2 | Primary dormancy-rep3 | Primary dormancy | Secondary dormancy | Vegetative growth rate | Flowering time | Population structure |
|---|---|---|---|---|---|---|---|---|---|---|---|---|
| 6600 | Aa-0 | Germany | 9.6 | 50.9 | 17.39 | NA | NA | 50.72 | 1.3 | 0.090105 | 67 | 0.509 |
| 22630 | Ag-0 | France | 1.5 | 45 | NA | 65 | 0.02 | 192.07 | NA | 0.071242 | 65.75 | 0.403 |
| 1656 | Alc-0 | Spain | -3.2 | 40.31 | NA | NA | NA | 264 | NA | 0.122722 | 67 | 0.459 |
| 22526 | Amel-1 | Netherlands | 5.8 | 53.1 | 32.77 | NA | NA | 72.32 | 1.54 | 0.064747 | 71.5 | 0.454 |
| 22626 | An-1 | Belgium | 4.5 | 51.4 | 3.5 | 1 | 0.12 | 7.14 | 0.99 | 0.073064 | 58.75 | 0.43 |
| 22519 | Ang | France | 0.6 | 47.5 | 4.93 | NA | NA | 107.44 | 0.12 | 0.083582 | 59.5 | 0.136 |
| 22520 | Ann-1 | France | 6.1 | 45.9 | 17.5 | NA | NA | 35.61 | 0.88 | 0.113886 | 65.75 | 0.217 |
| 22529 | Baa-1 | Netherlands | 6.1 | 51.3 | 55.46 | NA | NA | 98.77 | 2.21 | 0.099215 | 67.75 | 0.395 |
| 22633 | Bay-0 | Germany | 11 | 49 | 3.5 | 0 | 0.27 | 4.59 | 0.66 | 0.249604 | 58.5 | 0.555 |
| 6613 | Be-0 | Germany | 9 | 50 | 3.66 | NA | NA | 29.67 | 0.92 | 0.258749 | 64.75 | 0.559 |
| 22579 | Bil-7 | Sweden | 18.4 | 63.2 | 3.5 | 0 | NA | 6.42 | 1.25 | 0.056818 | 76.25 | 0.719 |
| 10184 | Bl-1 | Italy | 11 | 44 | 5.13 | NA | NA | 19.89 | 0.12 | 0.058957 | 59.75 | 0.231 |
| 10185 | Bla-10 | Spain | 2.6 | 41.5 | 25.87 | NA | NA | 58.08 | 0.27 | 0.115429 | 58.5 | 0.304 |
| 6645 | Blh-1 | Czech Republic | 16.5 | 48.6 | 38.61 | NA | NA | 45.77 | 0.16 | 0.199848 | 76 | 0.749 |
| 22551 | Boot | United Kingdom | -3.3 | 54.4 | 65.72 | NA | NA | 162.93 | NA | 0.191474 | 60.75 | 0.199 |
| 22590 | Bor-1 | Czech Republic | 16.5 | 49.2 | 3.5 | 34 | NA | 41.25 | 1.48 | 0.059935 | 67.25 | 0.799 |
| 22591 | Bor-4 | Czech Republic | 16.5 | 49.2 | 4.28 | 28 | NA | 40.47 | 1.98 | 0.124493 | 63 | 0.865 |
| 22628 | Br-0 | Czech Republic | 16.5 | 49 | NA | 73 | 0.1 | 160.66 | NA | 0.206641 | 61 | 0.592 |
| 6627 | Bs-1 | Switzerland | 7.4 | 47.4 | 17.36 | NA | NA | 64.06 | 0.07 | 0.094751 | 65.75 | 0.361 |
| 6094 | Bso-1a | France | 4.6 | 52.3 | 40.07 | NA | NA | 110.78 | NA | 0.059841 | 63.5 | 0.218 |
| 22656 | Bur-0 | Ireland | -8 | 53.5 | 3.5 | 0 | 0.19 | 3.52 | 0.88 | 0.098816 | 71.25 | 0.382 |
| 10257 | Byn | Norway | 10.1 | 63.3 | 3.5 | NA | NA | 4.13 | 0 | 0.049127 | 85 | 0.472 |
| 22620 | C24 | Portugal | -8.3 | 40.2 | 14.37 | 119 | 0.19 | 37.63 | 0.03 | 0.061173 | 59.75 | 0.335 |
| 6659 | Cal-0 | United Kingdom | -1.6 | 53.3 | 3.5 | NA | NA | 3.5 | 0.3 | 0.095204 | 72 | 0.377 |
| 10271 | Calamin | Belgium | NA | 50 | 17.07 | NA | NA | 109.58 | NA | 0.105172 | 64.75 | 0.558 |
| 6042 | Car-1 | Italy | 12.5 | 41.9 | 35.41 | NA | NA | 130.2 | NA | 0.099032 | 63 | 0.578 |
| 22523 | Cerv-1 | Italy | 12.5 | 41.9 | 40.5 | NA | NA | 102.25 | NA | 0.092917 | 68 | 0.45 |
| 22521 | Chat-1 | France | 1.3 | 48.1 | 12.33 | NA | NA | 150.36 | NA | 0.064677 | 61.25 | 0.417 |
| 22603 | CIBC-17 | United Kingdom | 0.6 | 51.4 | 33.15 | 55 | NA | 61.13 | 0.01 | 0.085809 | 68.5 | 0.549 |
| 22602 | CIBC-5 | United Kingdom | 0.6 | 51.4 | 17.28 | 52 | NA | 60.04 | NA | 0.159834 | 62.5 | 0.167 |
| 22639 | Ct-1 | Italy | 15 | 37.5 | 4.67 | 41 | 0.11 | 115.15 | NA | 0.086561 | 59.25 | 0.812 |
| 1116 | Dra-0 | Czech Republic | 16.3 | 49.4 | 4.25 | NA | NA | 22.19 | 0.73 | 0.137626 | 59 | 0.877 |
| 10038 | Driel | Netherlands | 5.8 | 52 | 6.96 | NA | NA | 24.94 | 0.15 | 0.068237 | 60.5 | 0.455 |
| 22572 | Eden-1 | Sweden | 18.1 | 62.6 | 3.5 | 2 | NA | 5.24 | 0.5 | 0.087789 | 79 | 0.654 |
| 22573 | Eden-2 | Sweden | 18.1 | 62.6 | 3.5 | NA | NA | 4.06 | 1.69 | 0.062693 | 82.5 | 0.631 |
| 22657 | Edi-0 | United Kingdom | -3 | 56 | 19 | 76 | 0.07 | 43.63 | 0 | 0.105011 | 71 | 0.611 |
| 6693 | Eil-0 | Germany | 12.6 | 51.5 | 5.15 | NA | NA | 26.34 | 0.96 | 0.123077 | 59 | 0.882 |
| 6088 | Ely-1a | United Kingdom | 0.3 | 52.4 | 22.15 | NA | NA | 34.64 | 0.03 | 0.078407 | 75 | 0.295 |

| | | | | | | | | | | | | | |
|---|---|---|---|---|---|---|---|---|---|---|---|---|---|
| 1637 | Ema-1 | United Kingdom | -0.2 | 51.3 | 61.2 | NA | NA | 94.19 | NA | 0.117075 | 61.25 | 0.086 |
| 1138 | En-2 | Germany | 8.8 | 50.2 | 11.32 | NA | NA | 78.47 | NA | NA | 63 | 0.799 |
| 22548 | Eri | Sweden | 15.4 | 56.5 | 3.5 | NA | NA | 11.66 | 0 | 0.063494 | 60.5 | 0.803 |
| 6699 | Es-0 | Finland | 25 | 60 | 12.32 | NA | NA | 47.03 | 0.06 | 0.145006 | 57.75 | 0.318 |
| 6700 | Est-0 | Estonia | 26 | 59 | 11.68 | NA | NA | 68.21 | NA | 0.181915 | 58.25 | 0.71 |
| 22629 | Est-1 | Estonia | 25.5 | 58.5 | 3.5 | 9 | NA | 36.94 | 2.28 | 0.065165 | 64.5 | 0.723 |
| 22645 | Fei-0 | Portugal | -8.3 | 40.55 | 10.79 | 23 | 0.07 | 73.19 | 0.29 | 0.048177 | 62.25 | 0.15 |
| 6705 | Fi-1 | Germany | 8 | 50.3 | 24.79 | NA | NA | 52.59 | 0.28 | 0.15035 | 58.75 | 0.454 |
| 1184 | Gd-1 | Germany | 10.8 | 53.6 | 24.17 | NA | NA | 76.42 | 1.12 | 0.164413 | 59 | 0.661 |
| 22608 | Got-7 | Germany | 9.8 | 51.4 | 39.7 | 61 | NA | 76.55 | 0.15 | 0.065799 | 84.5 | 0.427 |
| 22534 | Hey | Netherlands | 5.9 | 51.3 | 34.97 | NA | NA | 105.1 | 1.66 | 0.067229 | 66.5 | 0.267 |
| 22597 | HR-10 | United Kingdom | 0.6 | 51.4 | 29.31 | 43 | NA | 249.49 | NA | 0.187755 | 58.5 | 0.078 |
| 22596 | HR-5 | United Kingdom | 0.6 | 51.4 | 6.86 | 58 | NA | 90.49 | NA | 0.022797 | 66.5 | 0.103 |
| 10043 | Jea | France | 7.3 | 43.6 | 21.23 | NA | NA | 61.88 | NA | 0.045338 | 64.33 | 0.394 |
| 6752 | Ka-0 | Austria | 13.9 | 46.7 | 4.23 | NA | NA | 61.96 | 0 | 0.11431 | 60.25 | 0.574 |
| 6754 | Kil-0 | United Kingdom | -5.5 | 55.4 | 13.52 | NA | NA | 121.07 | NA | 0.10657 | 60.25 | 0.577 |
| 6045 | KL-PW-1 | Germany | 7 | 50.9 | 4.1 | NA | NA | 39.67 | 1.77 | 0.182751 | 60.75 | 0.426 |
| 1287 | Kn-0 | Lithuania | 23.7 | 54.7 | 3.5 | NA | NA | 10.22 | 0.15 | 0.044008 | 64.25 | 0.816 |
| 22618 | Ler-1 | Poland | 15.2 | 52.7 | 3.5 | 10 | 0.83 | 9.15 | 0.22 | 0.088498 | 61.25 | 0.815 |
| 22650 | LL-0 | Spain | 2.6 | 41.7 | 21.52 | 129 | 0.01 | 99.6 | NA | 0.04144 | 60.25 | 0.393 |
| 6784 | Lm-2 | France | 0.2 | 48 | 3.5 | NA | NA | 7.23 | 1.04 | 0.180164 | 57.5 | 0.193 |
| 22594 | Lp2-2 | Czech Republic | 16.5 | 49.3 | 11.45 | 13 | NA | 32.31 | 1.2 | 0.11761 | 60.5 | 0.685 |
| 22595 | Lp2-6 | Czech Republic | 16.5 | 49.3 | 3.5 | 6 | NA | 7.25 | 0.79 | 0.064857 | 63.75 | 0.813 |
| 22615 | Lz-0 | France | 3.5 | 46 | 36.08 | 56 | NA | 66.55 | 0.8 | 0.086364 | 65.75 | 0.38 |
| 6793 | Mh-1 | Poland | 20.3 | 53.6 | 3.5 | NA | NA | 264 | NA | 0.159998 | 59 | 0.712 |
| 22640 | Mr-0 | Italy | 9.5 | 44.5 | 61.2 | 122 | NA | 182.52 | NA | 0.07785 | 78.25 | 0.497 |
| 22635 | Mrk-0 | Germany | 9.5 | 49 | 16.71 | 17 | 0.09 | 34.59 | 0.74 | 0.119093 | 67.75 | 0.571 |
| 22636 | MZ-0 | Germany | 8.5 | 50.5 | 16.63 | 30 | 0.15 | 49.09 | 0.23 | 0.110865 | 62.5 | 0.671 |
| 22619 | Nd-1 | Germany | 10 | 51 | 11.84 | NA | 0.11 | 89.5 | NA | 0.052642 | 61 | 0.461 |
| 22599 | NFA-10 | United Kingdom | 0.6 | 51.4 | 9.09 | 5 | NA | 72.1 | NA | 0.112877 | 62.5 | 0.182 |
| 22598 | NFA-8 | United Kingdom | 0.6 | 51.4 | 7.53 | 38 | NA | 132.64 | 0.28 | 0.1469 | 59.75 | 0.085 |
| 1394 | No-0 | Germany | 13 | 51 | 3.5 | NA | NA | 10.75 | 0.8 | 0.103317 | 60.75 | 0.783 |
| 22643 | Nok-3 | Netherlands | 4 | 52.5 | 22.79 | 36 | 0.04 | 59.74 | 1.37 | 0.067242 | 71.5 | 0.444 |
| 22584 | Omo2-1 | Sweden | 15.3 | 56.2 | 20.64 | 73 | NA | 182.53 | NA | 0.043937 | 83.5 | 0.574 |
| 22585 | Omo2-3 | Sweden | 15.3 | 56.2 | 40.06 | 70 | NA | 58.23 | 0.03 | 0.143638 | 70.25 | 0.564 |
| 22649 | Pro-0 | Spain | -6 | 43.2 | 79.29 | 79 | 0.02 | 134.41 | 0.1 | 0.093241 | 58 | 0.206 |
| 22593 | Pu2-23 | Croatia | 18.1 | 42.5 | 3.5 | 28 | NA | 34.94 | 1.28 | 0.111549 | 65 | 0.914 |
| 22592 | Pu2-7 | Croatia | 18.1 | 42.5 | 13.24 | 33 | NA | 44.64 | 0.19 | 0.056905 | 68.25 | 0.845 |
| 22632 | Ra-0 | France | 3.4 | 46 | 49.98 | 64 | 0.05 | 105.46 | 1.06 | 0.062726 | 62 | 0.22 |
| 22610 | Ren-1 | France | -1.6 | 48.7 | 16.86 | 51 | NA | 39.53 | 0.18 | 0.042827 | 65.5 | 0.221 |
| 22611 | Ren-11 | France | -1.6 | 48.7 | 16.27 | 41 | NA | 54.97 | 0 | 0.080916 | 59 | 0.14 |
| 22524 | Rome-1 | Italy | 12.5 | 41.9 | 29.75 | NA | NA | 165.29 | NA | 0.102723 | 59.75 | 0.493 |
| 6917 | Sah-0 | Spain | 3.1 | 39 | 57.05 | NA | NA | 101.82 | 0.27 | 0.163547 | 76 | 0.622 |
| 1514 | Sav-o | Czech Republic | 13 | 49.8 | 3.5 | NA | NA | 21.77 | 2.26 | 0.155034 | 60.25 | 0.896 |
| 22646 | Se-0 | Spain | 2.5 | 41.5 | 18.57 | 123 | 0.09 | 59.28 | 0.18 | 0.058419 | 59.75 | 0.45 |
| 6077 | Sed-1 (Sid- | United Kingdom | -3.2 | 50.7 | 37.44 | NA | NA | 229.95 | NA | 0.082536 | 59 | 0.105 |

1)

| | | | | | | | | | | | | |
|---|---|---|---|---|---|---|---|---|---|---|---|---|
| 1504 | Sei-0 | Italy | 11.6 | 46.5 | 16.3 | NA | NA | 38.56 | 0.02 | 0.076746 | 62 | 0.589 |
| 1516 | Sf-2 | Spain | 2.2 | 42 | 12.6 | NA | NA | 61.38 | 0.89 | 0.185139 | 57.5 | 0.436 |
| 22582 | Spr1-2 | Sweden | 14.4 | 56.4 | 3.5 | NA | NA | 13.66 | 1.81 | 0.074689 | 81 | 0.559 |
| 22600 | Sq-1 | United Kingdom | 0.6 | 51.4 | 22.91 | NA | NA | 79.68 | NA | 0.063777 | 66.75 | 0.179 |
| 22601 | Sq-8 | United Kingdom | 0.6 | 51.4 | 17.5 | 28 | NA | 42.12 | 0.01 | 0.084515 | 60 | 0.365 |
| 1535 | St-0 | Sweden | 18 | 59 | 18.03 | NA | NA | 29.97 | 0.4 | 0.12492 | 67.33 | 0.502 |
| 10256 | Strand | Norway | 11.2 | 60.3 | 3.5 | NA | NA | 16.93 | 0.81 | 0.062556 | 71.75 | 0.546 |
| 22604 | Tamm-2 | Finland | 23.3 | 59.7 | 3.5 | 11 | NA | 11.97 | 0.21 | 0.085243 | 71.25 | 0.56 |
| 22605 | Tamm-27 | Finland | 23.3 | 59.7 | 3.5 | 6 | NA | 18.11 | 0.33 | 0.07202 | 72.5 | 0.691 |
| 6918 | Te-0 | Finland | 23.2 | 60.1 | 20.57 | NA | NA | 66.51 | 0.37 | 0.171372 | 71.25 | 0.588 |
| 10039 | Terlet | Netherlands | 6 | 52.1 | 3.91 | NA | NA | NA | NA | 0.087039 | 67.25 | 0.435 |
| 22537 | Tha-1 | Netherlands | 4.3 | 52.1 | 26.08 | NA | NA | 37.09 | 0.04 | 0.044591 | 71 | 0.459 |
| 22647 | Ts-1 | Spain | 3 | 41.5 | 18.93 | 129 | NA | 72.07 | NA | 0.141393 | 58 | 0.285 |
| 22648 | Ts-5 | Spain | 3 | 41.5 | 34.42 | 127 | 0.1 | 79.74 | NA | 0.12325 | 61.5 | 0.355 |
| 22518 | Tschag | Austria | 9.9 | 47.1 | 17.46 | NA | NA | 77.11 | 0.08 | 0.162568 | 62.75 | 0.691 |
| 10191 | Uk-2 | Germany | 7.6 | 48 | 55.87 | NA | NA | 75.12 | 1.01 | 0.211036 | 59 | 0.571 |
| 22587 | Ull2-3 | Sweden | 13.6 | 56.1 | 15.66 | 15 | NA | 75.55 | NA | 0.140898 | 64.75 | 0.509 |
| 22612 | Uod-1 | Austria | 14.7 | 48.1 | 12.67 | 32 | NA | 61.19 | 0.73 | 0.152667 | 65.75 | 0.602 |
| 22613 | Uod-7 | Austria | 14.7 | 48.1 | 16.72 | 46 | NA | 70.12 | 0.52 | 0.15326 | 65.5 | 0.584 |
| 10172 | Vil-0 | Spain | 3.8 | 42.8 | 42 | NA | NA | 51.5 | 0.11 | 0.097446 | 80.75 | 0.337 |
| 22644 | Wa-1 | Poland | 21 | 52.5 | 7.76 | 19 | 0.11 | 52.3 | 1.78 | 0.107253 | 59.25 | 0.78 |
| 22540 | Wag-1 | Netherlands | 5.7 | 52 | 3.5 | NA | NA | 17.28 | 0.6 | 0.078745 | 66.5 | 0.595 |
| 22622 | Wei-0 | Switzerland | 8.4 | 47.4 | 42.22 | 75 | 0.09 | 69.29 | 0.31 | 0.159259 | 57.25 | 0.414 |
| 10371 | Wha-2 | United Kingdom | -0.4 | 53 | NA | NA | NA | 180 | NA | 0.055824 | 65.5 | 0.091 |
| 22637 | Wt-5 | Germany | 9.5 | 52.5 | 42.55 | 65 | 0.02 | 125.69 | NA | 0.099735 | 65.5 | 0.599 |
| 22588 | Zdr-1 | Czech Republic | 16.5 | 49.2 | 10.03 | 20 | NA | 40.54 | 0.74 | 0.204754 | 58.75 | 0.889 |
| 22589 | Zdr-6 | Czech Republic | 16.5 | 49.2 | 3.5 | 16 | NA | 21.7 | 0.81 | 0.074019 | 67.5 | 0.816 |
| 1629 | Zu-1 | Switzerland | 8.5 | 47.3 | 8.25 | NA | NA | 66.55 | 0.19 | 0.085289 | 60.5 | 0.336 |
